# Veliparib in combination with radiotherapy for the treatment of *MGMT* unmethylated glioblastoma

**DOI:** 10.1186/s12967-017-1164-1

**Published:** 2017-03-17

**Authors:** Toni Rose Jue, Kyoko Nozue, Ashleigh J. Lester, Swapna Joshi, Lisette B. W. Schroder, Shane P. Whittaker, Sheri Nixdorf, Robert W. Rapkins, Mustafa Khasraw, Kerrie L. McDonald

**Affiliations:** 1 Cure Brain Cancer Biomarkers and Translational Research Group, Prince of Wales Clinical School, Adult Cancer Program, Lowy Cancer Research Centre, UNSW, Kensington, Australia; 2 Royal North Shore Hospital, St Leonards, NSW Australia

## Abstract

**Background:**

The *O* ^*6*^-*methylguanine methyltransferase* (*MGMT*) gene is frequently unmethylated in patients with glioblastoma (GBM), rendering them non-responsive to the standard treatment regime of surgery followed by concurrent radiotherapy (RT) and temozolomide. Here, we investigate the efficacy of adding a PARP inhibitor, veliparib, to radiotherapy to treat *MGMT* unmethylated GBM.

**Methods:**

The inhibition of PARP with veliparib (ABT-888), a potent and orally bioavailable inhibitor in combination with RT was tested on a panel of patient derived cell lines (PDCLs) and patient-derived xenografts (PDX) models generated from GBM patients with *MGMT* unmethylated tumors.

**Results:**

The combination of veliparib and RT inhibited colony formation in the majority of PDCLs tested. The PDCL, RN1 showed significantly reduced levels of the homologous repair protein, Mre11 and a heightened response to PARP inhibition measured by increased apoptosis and decreased colony formation. The oral administration of veliparib (12.5 mg/kg, twice daily for 5 days in a 28-day treatment cycle) in combination with whole brain RT (4 Gy) induced apoptosis (Tunel staining) and decreased cell proliferation (Ki67 staining) in a PDX of *MGMT* unmethylated GBM. Significantly longer survival times of the PDX treated with the combination treatment were recorded compared to RT only or veliparib only.

**Conclusions:**

Our results demonstrate preclinical efficacy of targeting PARP at multiple levels and provide a new approach for the treatment of *MGMT* unmethylated GBM.

## Background

Glioblastoma (GBM) is a uniformly lethal disease that has had few therapeutic advances over the past century. The standard treatment for GBM consists of radiotherapy (RT) combined with temozolomide (TMZ) chemotherapy followed by at least six cycles of TMZ. The median survival is less than 15 months [1]. Survival is significantly worse for patients whose tumor is unmethylated at the *O* ^*6*^-*methylguanine*-*DNA methyltransferase* (*MGMT*) promoter because they do not respond to DNA damaging therapy [2]. In a study by Hegi and colleagues, a survival benefit was seen in patients with *MGMT* methylated tumors: median survival 23.4 months compared to 12.6 months in those with non-methylated tumors treated with the concurrent RT and TMZ regimen [2]. Given the response to TMZ is limited, trials to find better therapies for GBM patients, particularly for those with an unmethylated *MGMT* promoter are imperative.

Inhibition of DNA repair by PARP inhibitors during RT and chemotherapy has been explored both pre-clinically and clinically in numerous solid cancers including breast, ovarian, rectal, prostate, and lung cancer [3–6]. Sensitivity to PARP inhibitors is mediated by mutations in the *BRCA1* and two genes, which result in the defective function of the homologous recombination (HR) pathway [7–9]. PARP inhibitors act through synthetic lethality by targeting the base excision repair (BER) pathway. Disruption of both HR and BER pathways lead to cell death. Clinically, the PARP inhibitor veliparib (ABT-888; AbbVie) is being investigated in Phase three studies of several cancers including lung (NCT02264990), triple negative breast cancers (NCT02032277) and *HER2* negative *BRCA1/2* deficient breast cancers (NCT02163694).

Sensitivity to PARP inhibition has been observed in cancers that do not harbor *BRCA1/2* deficiencies. Many other gene mutations, commonly found in cancer, result in recombination defects and hence the likely sensitivity to PARP inhibitors. Mutations in genes such as *PTEN* [10, 11], *ATM* [12, 13], *PALB2* [14], *CHEK2* [13, 15, 16], *FANCA* [15] and *HDAC2* [16] have been implicated in patient response to PARP inhibitors. However, it is noteworthy that >50 genes, many mutated in cancer, can confer sensitivity to PARP inhibitors [4].

For the treatment of GBM, studies have focused on the use of PARP inhibitors as radio- or chemo-sensitizers [11, 17–20]. In a study using veliparib in combination with TMZ, sensitivity to TMZ was significantly improved in both *MGMT* promoter methylated and unmethylated cell lines [19]. However this chemo-sensitizing effect was not observed in vivo when *MGMT* unmethylated lines were intracranially injected into immunocompromised mice, suggesting that only GBM that are *MGMT* methylated confer a benefit to combination treatment. A recent study evaluated the triple combination therapy consisting of veliparib given concurrently with RT and TMZ in a genetically engineered mouse model whose induced GBM was sensitive to TMZ [21]. The study reported statistically significant improvement in overall survival of the mice treated with the triple combination; however this is not clinically relevant as the triple combination treatment incurs significant toxicity. A clinical trial evaluating veliparib in combination with adjuvant TMZ for newly diagnosed GBM patients with *MGMT* promoter methylation following standard RT is currently enrolling patients (NCT02152982).

The combination of PARP inhibitors with radiation therapy has also been trialed pre-clinically [22–24]. Venere and colleagues provided compelling evidence that GBM-initiating cells (GICs) express high levels of PARP1. These cells are typically resistant to RT [24]. Significant loss of viability in vitro was observed when the PARP inhibitor, olaparib was combined with RT compared to either agent alone. Tumors were completely abolished with this combination when tested in vivo [24].

Treatment for *MGMT* unmethylated patients is a significant unmet need and new treatments are urgently needed to (1) replace TMZ as a therapy and/or (2) to improve and sensitize tumors to the current standard of therapy. We have developed a large series of patient derived cell lines that are *MGMT* unmethylated. We proposed to treat these cell lines with a combination of RT and veliparib to demonstrate that PARP inhibitors can be effective as radiosensitizers both in vitro and in vivo. In addition, we explored identification of biomarkers of response to the veliparib/RT combination so that this can be translated back to the clinic for patient stratification.

## Methods

### Drugs

Veliparib (ABT-888) was provided via a research agreement with AbbVie. Temozolomide (TMZ) was purchased from Sigma Aldrich.

### Patient derived cell lines (PDCLs)

The PDCLs, G13, G18, G28, G56, G54, G57 and G89, were generated from fresh GBM tumor tissue collected at time of surgery in accordance with appropriate approved institutional review board protocols. All patients signed a consent form prior to collection. Within 10 min of collection from surgery, tumor tissue was dissociated using the GentleMACS™ Dissociator (Miltenyi Biotec) in the presence of Accutase^®^ solution (Sigma-Aldrich). Following addition of trypsin inhibitor (1:1 ratio; Sigma-Aldrich), cells were passed through a 100 µm Falcon™ cell strainer (Corning Inc.) to collect a suspension of single cells. Centrifugation was performed at 300 rcf for 5 min at room temperature. Cell pellets were resuspended in RHB-A medium (Clontech Laboratories Inc.) supplemented with human epidermal growth factor (20 ng/mL; Sigma-Aldrich) and human fibroblast growth factor—basic (20 ng/mL; Sigma-Aldrich), and were seeded into tissue culture flasks coated with a layer of BD Matrigel™ basement membrane matrix (1:100 in PBS; BD Biosciences). Cells were maintained in a 37 °C, 5% CO_2_ incubator (Thermo Fisher Scientific). Initially, cultures were left undisturbed for 5 days prior to media replacement to allow for cell attachment. Following this period, cell lines underwent a media change twice weekly and were passaged once a confluency of 80% was reached. The PDCLs, WK1 and RN1 were a generous gift from Dr Bryan Day, QIMR Berghofer. WK1 and RN1 were also maintained in RBH-A medium.

### MGMT testing

All PDCLs were screened for *MGMT* promoter methylation using a pyrosequencing assay as described previously [25]. Briefly, DNA was extracted from the cell lines and converted with sodium bisulfite using the EZ Methylation-Gold kit (as per the manufacturers instructions; Zymo). The methylation-unbiased pyrosequencing assay was performed using the PyroMark MGMT kit (Qiagen) on the PSQ24 MA system (Qiagen) and interrogated five individual CpG sites within exon one near the *MGMT* transcription start site for methylation [25] PyroMark CpG software (Qiagen) was used to quantify the levels of methylation. GBM cell lines were scored as methylation positive by pyrosequencing if all five CpG sites had methylation values of 9% or higher [25]. Chemically methylated and non-methylated genomic DNA was used as positive and negative controls, respectively (Millipore).

### Colony formation assay

Colony formation assays for the PDCLs were performed as previously described [26]. PDCLs were plated in triplicate in 6-well plates and incubated overnight. The cells were treated with vehicle control (DMSO) or veliparib (10 μM) in supplemented RBH-A medium. Radiation was delivered using a self-contained X-ray system (X-RAD 320). Plates were incubated for 2 weeks undisturbed. Colonies were gently washed with PBS followed by staining and fixation with crystal violet solution (0.5% in H_2_O:methanol, 1:1) for 15 min. Stained colonies consisting of >50 cells were counted and the number was recorded. Plating efficiency was calculated as the number of colonies counted divided by the number of cells seeded and normalized to the average plating efficiency of untreated samples. The average of these values was reported as “percentage of cells survived compared to the control.”

### Cell proliferation assay

The optimum cell density of each PDCL was established using the MTS, CellTiter 96^®^ Aqueous Assay (Promega^®^) and viability was measured 8 days post-treatment. PDCLs were treated with increasing concentrations of veliparib (1–10 μM); TMZ (100–300 μM) and/or radiation (1–4 Gy) to determine the cytotoxic effects of the chemotherapeutic drugs, and the half-maximal inhibitory concentration (IC_50_).

### Flow cytometry

PDCLs were seeded in 6-well plates for 24-h. Cells were treated with veliparib (10 μM) 2 h prior to RT (4 Gy). The plates were incubated for a further 72-h. Fluoroscein isothiocyante (FITC)-conjugated Annexin V and propidium iodide (PI) stains (Roche) were used to measure apoptotic cell death. In brief, treated cells were harvested and the cell pellet was stained with Annexin-V-FITC and/or PI diluted in incubation buffer (1:50) following the manufacturer’s protocol. Apoptosis was measured using flow cytometry (BD FACScanto™ II).

### Western blot

Protein was extracted from untreated PDCLs using cell lysis buffer (10 mM Tris–Cl pH 7.4, 100 mM NaCl, 1 mM EDTA pH 8.0, 1 mM NaF, 20 mM Na_4_P_2_O_7_, 0.1% SDS, 0.5% sodium deoxycholate, 1% Triton X-100, 10% Glycerol, Milli-Q water) and complete, mini, EDTA-free protease inhibitor tablets (Sigma Aldrich). Western blots were probed with antibodies against Mre11 (Mouse monoclonal [12D7], 1:500) and Rad50 (Mouse monoclonal [13B3/2C6], 1:500). To control for protein loading, membranes were probed with EIF4E (Rabbit monoclonal [C46H6], 1:1000).

### In vivo experiments

Female athymic nude mice (Balb/c; 8–9 weeks of age) were intracranially injected with 2 × 10^5^ RN1 PDCLs stereotactically in the right caudate putamen using the coordinates: 1 mm anterior, 1.5 mm lateral, and 3.0 mm below the bregma. To monitor tumor growth, animals were humanely euthanized at the following time-points: 40, 45, 50 and 60 days. Mouse brains were fixed in formalin and embedded in paraffin. H&E stains of the brains revealed tumor growth by day 45, indicating the time of commencement of treatment. Mice were randomly assigned to four groups; (1) untreated control (n = 5); (2) veliparib only (12.5 mg/kg, twice daily gavage for 5 days in a 28-day treatment cycle) (n = 5); (3) radiation treatment (total of 4 Gy over 2 days) (n = 5) and (4) veliparib combined with radiation (n = 7). Whole brain radiation was delivered using a self-contained X-ray system (X-RAD 320). During RT, mice were placed in a customized lead box to shield the body to allow radiation to be delivered directly to the entire brain. The total radiation dose administered was 4 Gy at a clinically relevant 2 Gy/fraction schedule on two consecutive days. Two cycles of veliparib were administered to the mice, before endpoint was reached.

Mice were euthanized when they exhibited symptoms indicative of significant compromise to neurologic function and/or a greater than 20% body weight loss. Animal survival was defined as the time taken from tumour injection until euthanasia and survival curves were established using the Kaplan–Meier estimator.

### Immunohistochemistry

At the time of euthanasia, all brains were resected, formalin fixed and embedded in paraffin. Sections were cut at 4 μm and mounted on ultrafrost slides. All sections were deparaffinized in xylene and rehydrated in PBS using an ethanol gradient and a heat-mediated antigen retrieval step was performed using the Target Retrieval Solution (Dako, S1700) at 95 °C for 20 min. Sections were stained with an antibody against the proliferative marker, Ki67 (Monoclonal mouse anti-human Ki67 [MIB-1 clone] 1:100 Dako). A biotinylated polyclonal goat anti-mouse immunoglobulin (1:300 Dako) was used as the secondary antibody.

Sections were also stained using the in situ Cell Death Detection Kit, POD (Roche) to measure the degree of DNA strand breaks (TUNEL technology), a hallmark of apoptosis.

Both Ki67 and TUNEL positive tumour cells (nuclear staining) were counted in five random fields (20× magnification) per section and presented as percentage positive staining.

### Statistics

Statistical significance was calculated with GraphPad Prism Software utilizing a one- or two-way ANOVA with a Bonferroni’s post hoc test, Students *t* test or log-rank (Mantel-Cox) test, where appropriate (GraphPad Software Inc., San Diego, CA, USA). Data are represented as the mean ± S.D.

## Results

### *MGMT* unmethylated PDCLs respond to veliparib in combination with radiation treatment (RT)

Pyrosequencing to determine *MGMT* promoter methylation was performed on 15 individual GBM PDCLs. Approximately nine PDCLs (RN1, WK1, G13, G18, G28, G56, G54, G57 and G89) were confirmed to be *MGMT* unmethylated. These cell lines were selected for further analysis.

**Fig. 1:**
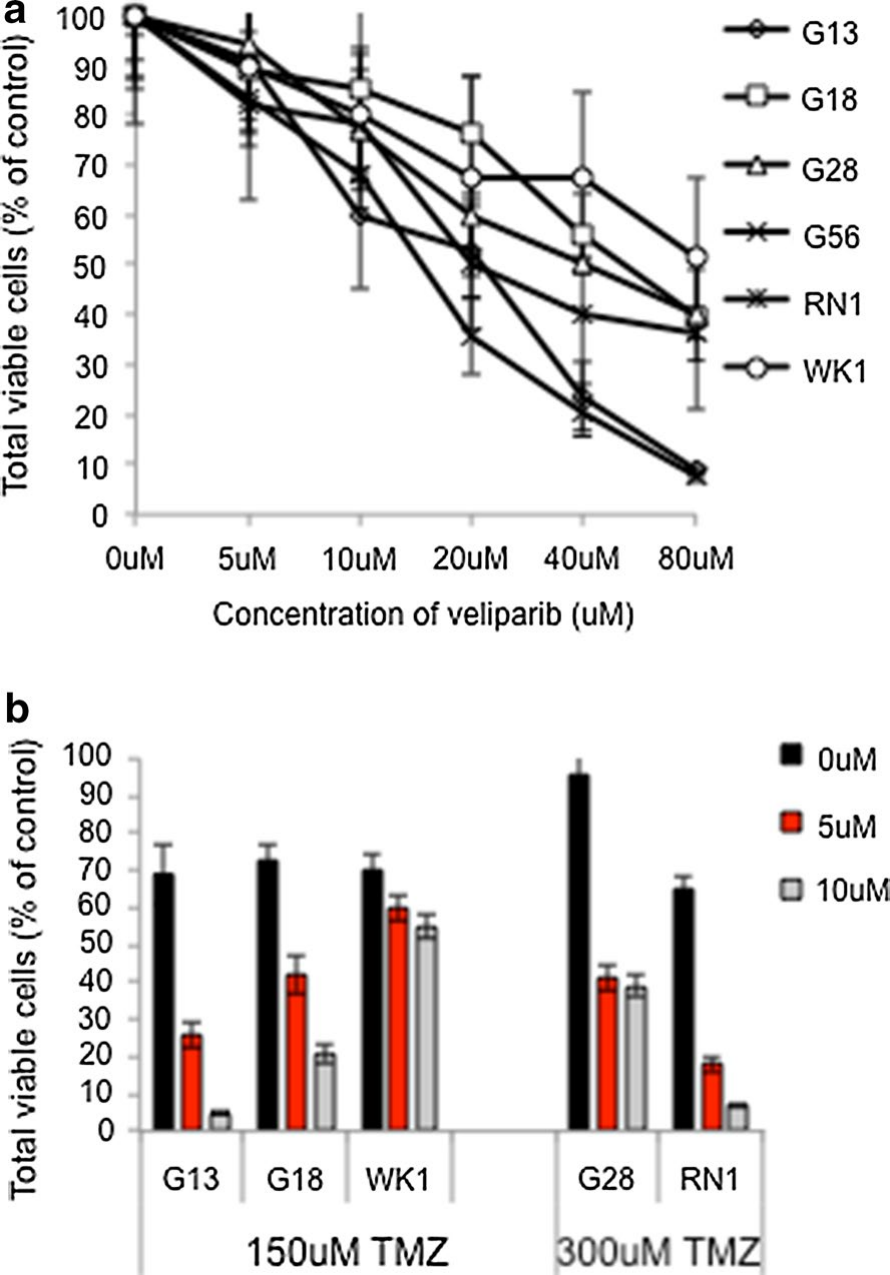
Effect of veliparib on *MGMT* unmethylated patient derived cell lines. **a** Dose-response curves of PDCLs treated with veliparib compared to untreated cells. **b** Combination of veliparib (0, 5 or 10 μM) with temozolomide (TMZ; 150 or 300 μM). *Bar graphs* represent percentage viability of cells after 8 days of treatment. *Error bars* represent the standard deviation of three individual experiments. Significance determined by student *t*-test where ***(p < 0.001)

Six *MGMT* unmethylated PDCLs (RN1, WK1, G13, G18, G28 and G56) were treated with different concentrations of veliparib for 72 h to determine the inhibitory concentration to kill 50% of cells (IC_50_) (Fig. 1a). High doses of veliparib were required to achieve IC_50_ for all PCDLs (18 up to 80 μM) indicating that veliparib would not be effective in GBM as a monotherapy. We combined veliparib (5 and 10 μM) with IC_50_ doses of TMZ (150 μM for G13, G18 and WK1; 300 μM for G28 and RN1) (Fig. 1b). In agreement with previous studies [19], significant synergism between the two drugs was observed in all cell lines, except for WK1. Gupta and colleagues reported potent efficacy when veliparib was combined with TMZ in vitro, however these results did not translate to an effect in vivo [19].

**Fig. 2:**
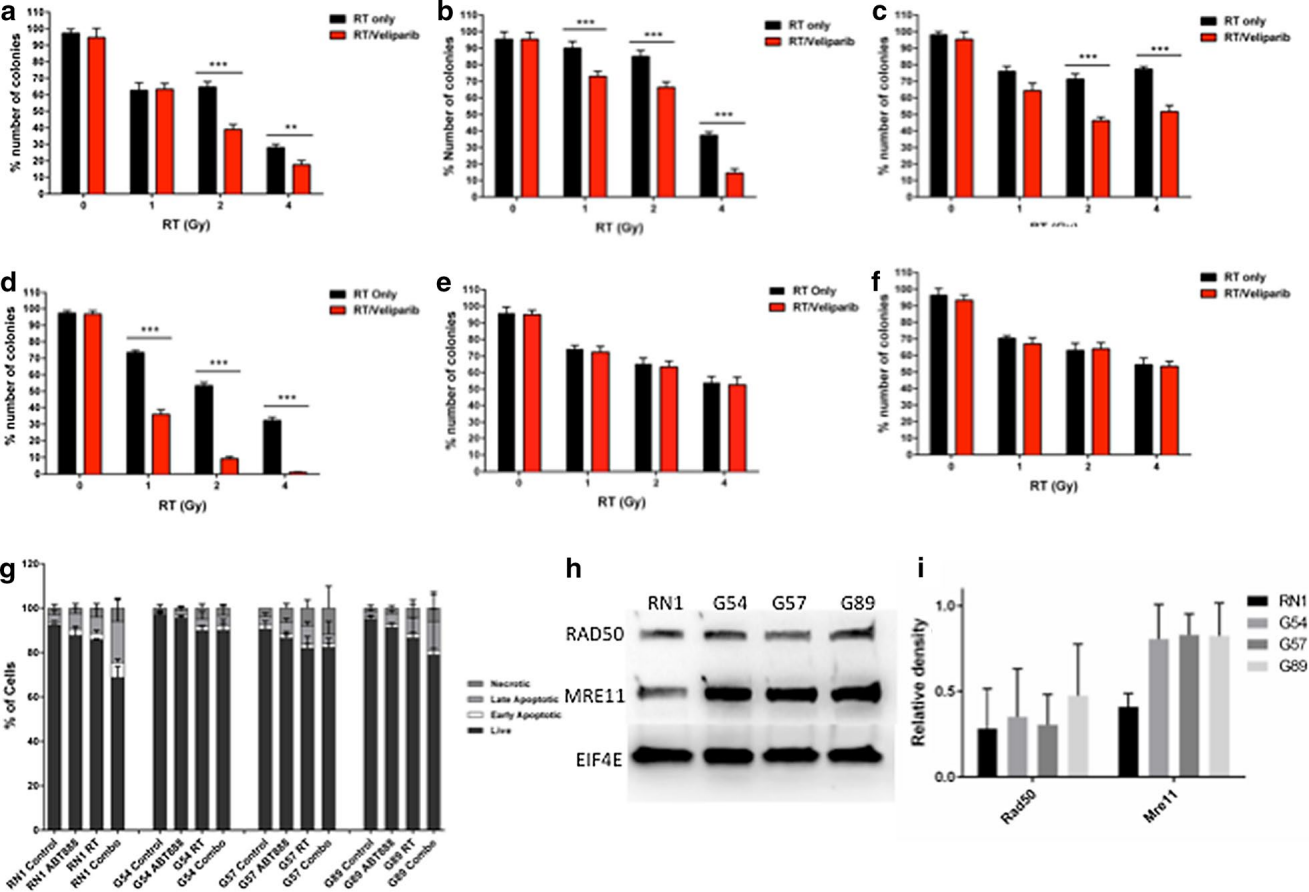
Combination of veliparib and radiotherapy. Patient derived cell lines were treated with veliparib (10 μM) for 2 h before irradiation with 0, 1, 2 and 4 GY. Cells were seeded in wells and the ability to form clones was monitored over a 10-day period. **a** G18; **b** WK1; **c** G53; **d** RN1; **e** G54 and **f** G57. *Bar graphs* represent the percentage of clones counted with respect to the control. **g** Veliparib and radiotherapy induce apoptotic cell death in patient derived cell lines. RN1, G54, G57 and G89 cells were treated with veliparib (10 μM) for 2 h before irradiation with 2 GY. **h** Protein expression of Rad50 and Mre11 were assessed in untreated PDCLs, RN1, G54, G57 and G89. **i** Quantification of Rad50 and Mre11 protein expression for each PDCL. *Error bars* represent the standard deviation of three individual experiments. Significance determined by student *t*-test where ***(p < 0.001); **(p < 0.01); *(p < 0.05)

Our primary objective was to determine if *MGMT* unmethylated cells responded to a combination of RT and veliparib. The effect on cell viability was measured by the ability of cells to form colonies. Clonogenic assays were performed in triplicate for G18, WK1, G53, RN1, G54 and (Fig. 2a–f). Cells were treated with a low dose of veliparib (10 μM) and irradiated with 1, 2 or 4 Gy 2 h later and incubated for a further 10 days before the counting of colonies and presented as a percentage of the control. For most of the cell lines, the ability to form colonies was significantly impeded when treated with veliparib and radiotherapy (2 Gy) (Fig. 2a–d). RN1 (Fig. 2d) was the most sensitive cell line to the combined treatment of veliparib and RT (1–4 Gy), recording over 50% colony inhibition. In contrast, two PDCLs (G54 and G57) showed little response to any of the treatment arms (Fig. 2e, f).

### Combination of veliparib and RT induces apoptosis

The PDCLs RN1, G54, G57 and G89 were treated with RT (4 Gy) and veliparib (10 μM) for 3 days. Media and drug were replenished daily. Cells were harvested and stained with Annexin V and propidium iodide to measure apoptotic cell death. Figure 2g summarizes the percentage of cells that are either (a) live; (b) in early apoptosis; (c) in late apoptosis or (d) necrotic. Treatment of RN1 with the combination of veliparib and RT showed a significant increase in the percentage of cells undergoing early–late apoptosis compared to control, veliparib only and RT only. The percentage cells positive for apoptosis was 33.8% compared to 6.6, 13.0 and 14.0% for control, veliparib only and RT only, respectively (p < 0.001). There was a slight increase in percentage cells undergoing apoptosis for G57 when treated with the combination of veliparib and RT, however this did not reach significance. No significant changes in apoptosis were detected for the resistant cell lines, G54 and G89 when treated with the combination.

### Loss of the MRN complex confers sensitivity to veliparib and radiotherapy

We performed Western blot analysis to examine the protein expression of Mre11 and Rad50 in the four PDCLs, RN1, G54, G57 and G89. Mre11 and Rad50 form a complex with the Nbs protein and play a key role in the sensing, processing and repair of double strand breaks. Significantly lower Mre11 protein expression was detected in RN1 (Fig. 2h, i) which was the most sensitive cell line to the combination of veliparib and RT, implicating loss of the MRN complex as a possible mechanism for sensitivity.

### Combining veliparib and RT leads to longer survival in an orthotopic model

**Fig. 3:**
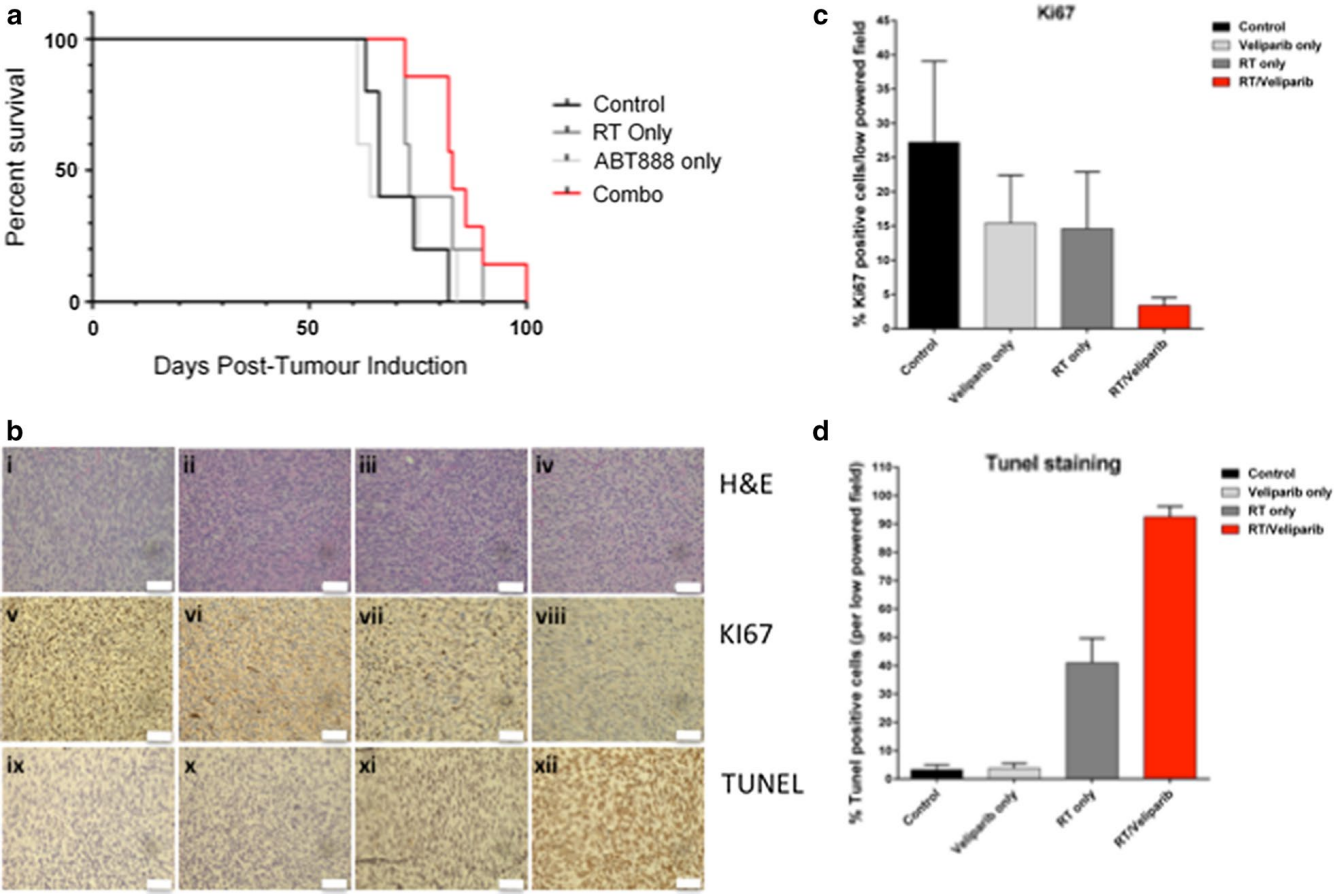
Treatment of a MGMT unmethylated PDX mouse model with oral veliparib and radiotherapy. **a** Kaplan–Meier survival curves demonstrate a significant survival advantage when mice were treated with the combination of veliparib and radiotherapy. The treatment schedule consisted of concomitant veliparib (12.5 mg/kg, twice daily gavage for 5 days in a 28-day cycle) and radiotherapy (total of 4 Gy given over 2 days). **b** Histology of resected tumors post-treatment. The *top panel* displays the H&E, *middle panel* are stained for Ki67 while the *bottom panel* are stained for Tunel. (*i*, *v* and *ix* are tumors resected from control mice; *ii*, *vi*, *x* are tumors resected from veliparib monotherapy treatment; *iii*, *vii*, *xi* are tumors resected from radiotherapy only treatment and *iv*, *viii*, *xii* are tumors resected from the combination of veliparib and radiotherapy. Representative images of three mice per group are shown). **c** Quantitative analysis of the Ki67 staining in all treatment groups; **d** Quantitative analysis of the Tunel staining in all treatment groups. *Error bars* represent the standard deviation of three individual experiments. Significance determined by student *t*-test where ***(p < 0.001); **(p < 0.01); *(p < 0.05)

We evaluated the combination treatment further by intracranially injecting balb/c nude mice with RN1 cells. We allowed the tumor to develop for 45 days (confirmed by histology) before commencing treatment. Consistent with observed chemo-radiosensitization in vitro, the combination of veliparib and RT resulted in significant survival benefit in the RN1 mouse model compared to veliparib alone or RT alone (Fig. 3a). Median survival for mice treated with the combination was 83 days compared to veliparib alone (64 days) and RT alone (73 days) (LogRank p value = 0.042).

Mouse brains were resected from euthanized animals, formalin fixed and paraffin embedded and sectioned for immunohistological staining with the proliferation marker, Ki67 and the apoptosis marker, Tunel (Fig. 3b–d). Examination of the H&E sections confirmed GBM had formed in all cases, hallmarked by increased mitotic bodies, necrosis and microvascular proliferation (Fig. 3b). Interestingly we noted that the number of mitoses per low powered field was markedly reduced in the brains from mice treated with the combination of veliparib and RT. This observation was confirmed by the percentage of cells stained positive for Ki67 (Fig. 3b, c). On average, less than 5% of tumor cells stained positive for Ki67 when treated with the combination compared to 12 and 14% for RT alone and veliparib alone, respectively. To our surprise, nuclei positivity for Tunel in combination treated tumors was dramatic, with over 90% of nuclei staining positive for Tunel (Fig. 3b, d). Tunel positive nuclei were also observed in tumors treated with RT only (40%) with very few cells positive in the veliparib only or untreated control tissues.

## Discussion

The inhibition of PARP has been shown to be efficacious when combined with TMZ, however, enhanced survival was only observed in *MGMT* methylated GBM [19]. These results formed the foundation for the clinical trial evaluating veliparib in combination for newly diagnosed *MGMT* methylated GBM patients (NCT02152982). However, patients diagnosed with *MGMT* unmethylated GBM have a worse prognosis are more effective therapies are urgently needed. We, herein, proposed the question: could veliparib sensitize *MGMT* unmethylated tumors to RT?

We utilized our GBM patient-derived cell lines, which were all tested for *MGMT* and found to be unmethylated to address this question. As with previous studies, we found veliparib as a monotherapy did not have an appreciable effect on cell viability in vitro or on tumor growth in vivo. However, when given in a concomitant fashion with RT, apoptotic cell death was induced, the ability to form colonies was reduced and the overall survival of our orthotopic model significantly increased.

Concomitant veliparib and RT has been tested previously in a subcutaneous model, however this is the first study demonstrating a significant effect of the combination treatment in an orthotopic model. We demonstrated an extension of 10 days in survival times for mice treated with the combination when compared to RT as a monotherapy. Tumors were significantly reduced in size and demonstrated high levels of apoptosis (detected by Tunel staining) and a lower proliferation index (measured by Ki67 staining). This positive signal has provided the foundations for a clinical trial in Australia: A Randomised Phase II study of veliparib and RT with adjuvant TMZ and veliparib versus standard RT and TMZ followed by TMZ in patients with newly diagnosed GBM with unmethylated O(6)-methylguanine-DNA methyltransferase (The VERTU study; ANZCTR: U1111-1167-6365). Over 20 patients have been randomized and the combination treatment has been well tolerated. The study aims to enroll 120 patients.

An important observation was made in the in vitro studies of veliparib and RT. Not all of the *MGMT* unmethylated cell lines responded favorably to treatment. The PDCLs, G54 and G89 did not show significant impediment of their ability to form colonies and the rate of apoptosis was not significantly different to the control cells. We tested the protein expression levels of Mre11 and Rad50. Together with Nsb1, they form the MRN complex that is involved in the detection and repair of DNA double-strand breaks (DSBs). The most sensitive PDCL, RN1 demonstrated significantly reduced expression of Mre11 protein. Reduced Mre11 expression has been implicated in mediating sensitivity to PARP inhibition in endometrial and colorectal cancers [27, 28]. Colorectal cancer cells harboring biallelic *Mre11* mutations were more sensitive to the PARP inhibitor, LT-626 and stable overexpression or knock-down of Mre11 in cell lines correlated with sensitivity [28]. We have not screened our PDCLs for mutations in the *Mre11* gene however, the clinical samples collected as part of the VERTU trial will be tested for both mutations and protein expression.

## Conclusion

We have shown that concomitant veliparib and RT is an effective treatment for *MGMT* unmethylated GBM. One caveat however, will be to screen patients on trial for biomarkers predictive of sensitivity as we have shown that not all patients will respond to the treatment.

## Abbreviations

GBM: glioblastoma
MGMT: O^6^-methylguanine methyltransferase
PARP: poly (ADP-ribose) polymerase
RT: radiotherapy
TMZ: temozolomide
PDCL: patient derived cell line
PDX: patient derived xenografts
HR: homologous repair
